# Cell-Based HIF1α Gene Therapy Reduces Myocardial Scar and Enhances Angiopoietic Proteome, Transcriptomic and miRNA Expression in Experimental Chronic Left Ventricular Dysfunction

**DOI:** 10.3389/fbioe.2022.767985

**Published:** 2022-05-12

**Authors:** Edit Gara, Sang-Ging Ong, Johannes Winkler, Katrin Zlabinger, Dominika Lukovic, Bela Merkely, Maximilian Y. Emmert, Petra Wolint, Simon P. Hoerstrup, Mariann Gyöngyösi, Joseph C. Wu, Noemi Pavo

**Affiliations:** ^1^ Heart and Vascular Centre, Semmelweis University, Budapest, Hungary; ^2^ Division of Cardiology, Department of Internal Medicine II, Medical University of Vienna, Vienna, Austria; ^3^ Stanford Cardiovascular Institute, Stanford, CA, United States; ^4^ Institute for Regenerative Medicine (IREM), University of Zurich, Zurich, Switzerland; ^5^ Department of Cardiothoracic and Vascular Surgery, German Heart Center Berlin, Berlin, Germany; ^6^ Department of Cardiovascular Surgery, Charité Universitätsmedizin Berlin, Berlin, Germany

**Keywords:** chronic ischemic heart failure, porcine infarction model, mesenchymal stem cells, minicircle vector, HIF1α, adverse remodeling

## Abstract

Recent preclinical investigations and clinical trials with stem cells mostly studied bone-marrow-derived mononuclear cells (BM-MNCs), which so far failed to meet clinically significant functional study endpoints. BM-MNCs containing small proportions of stem cells provide little regenerative potential, while mesenchymal stem cells (MSCs) promise effective therapy *via* paracrine impact. Genetic engineering for rationally enhancing paracrine effects of implanted stem cells is an attractive option for further development of therapeutic cardiac repair strategies. Non-viral, efficient transfection methods promise improved clinical translation, longevity and a high level of gene delivery. Hypoxia-induced factor 1α is responsible for pro-angiogenic, anti-apoptotic and anti-remodeling mechanisms. Here we aimed to apply a cellular gene therapy model in chronic ischemic heart failure in pigs. A non-viral circular minicircle DNA vector (MiCi) was used for *in vitro* transfection of porcine MSCs (pMSC) with HIF1α (pMSC-MiCi-HIF-1α). pMSCs-MiCi-HIF-1α were injected endomyocardially into the border zone of an anterior myocardial infarction one month post-reperfused-infarct. Cell injection was guided *via* 3D-guided NOGA electro-magnetic catheter delivery system. pMSC-MiCi-HIF-1α delivery improved cardiac output and reduced myocardial scar size. Abundances of pro-angiogenic proteins were analyzed 12, 24 h and 1 month after the delivery of the regenerative substances. In a protein array, the significantly increased angiogenesis proteins were Activin A, Angiopoietin, Artemin, Endothelin-1, MCP-1; and remodeling factors ADAMTS1, FGFs, TGFb1, MMPs, and Serpins. In a qPCR analysis, increased levels of angiopeptin, CXCL12, HIF-1α and miR-132 were found 24 h after cell-based gene delivery, compared to those in untreated animals with infarction and in control animals. Expression of angiopeptin increased already 12 h after treatment, and miR-1 expression was reduced at that time point. In total, pMSC overexpressing HIF-1α showed beneficial effects for treatment of ischemic injury, mediated by stimulation of angiogenesis.

## Introduction

Ischemic cardiovascular diseases, chronic, and end-stage heart failure are a huge socioeconomic burden on cardiovascular care (Elgendy, Mahtta, and Pepine 2019). Cell-based or cell-free regenerative treatment are thought to be effective in regenerative cardiology (Cahill, Choudhury, and Riley 2017; Ward, Abadeh, and Connelly 2018). Reliable meta-analyses showed that implantation was safe and feasible and tumorigenic adverse events did not occur (Gyongyosi et al., 2015; Gyöngyösi et al., 2016), though most clinical studies were performed on a relatively small number of patients. Most early pre-clinical and clinical trials on cardiac regeneration investigated the efficiency of cardiac administration of bone-marrow-derived mononuclear cells (BM-MNCs) and mesenchymal stem cells (MSCs). Despite safety and feasibility of BM-MNCs transplantation, a comprehensive meta-analysis of intracoronary administered BM-MNCs in patients with acute myocardial infarction (AMI) failed to show a clinically relevant efficacy (Gyongyosi et al., 2015). Reasons for the lack of clinically significant therapeutic effect of BM-MNCs are 1) they comprise of a heterogenous population of mature cells with limited proliferation capacity, 2) BM-MNCs lack a sufficient population of stem cells with regenerative capacity (Sugihara et al., 2007; Wang et al., 2020). BM-MNCs heterogenous cell population mostly contain hematopoietic precursor cells, hemangioblasts and endothelial progenitors with no capacity to multipotent differentiation. Although BM-MNCs stain positive for CD133 stem cell marker, their proliferative potential is limited to tissue-committed growth within hematopoietic development lacking transdifferentiating activity (Park, Ma, and Choi 2005; Ishikawa et al., 2006). Adequate procedures for isolating MSCs from the bone marrow are a decisive factor for generating robust preclinical data and for enhancing cardiac repair (Smits et al., 2005). MSCs are considered as being paracrine factories (Phinney and Pittenger 2017; Keshtkar, Azarpira, and Ghahremani 2018), and they modulate epigenetic changes by “hit-and-run” mechanisms multiplying their paracrine potency (Jungwirth et al., 2018; Park et al., 2018). Pluripotent stem cells-derived cardiovascular cells promise personalized therapy, yet concerns of safety and immunogenicity exist (Fernandez-Aviles et al., 2017; Madonna et al., 2019).

Realizing the pitfalls of cell therapy in cardiac regeneration, cardiac gene therapy is coming into the forefront. Animal models of cardiac gene therapy by using intracellular plasmid delivery suggested benefits of such approaches, as adenoviral delivery of vascular endothelial growth factor (VEGF) increased myocardial perfusion in a porcine ischemic myocardium (Hassinen et al., 2016). However, similar to cell-based clinical trials, cardiac gene therapy did not yet lead to a breakthrough in the treatment of chronic ischemic human heart (Yuan et al., 2018). To achieve clinically relevant success, timing of delivery, type and source of genes for cardiac therapy, furthermore, tracking methods need to be optimized (Grochot-Przeczek, Dulak, and Jozkowicz 2013; Scarfe et al., 2017; Tompkins et al., 2018).

**FIGURE 1:**
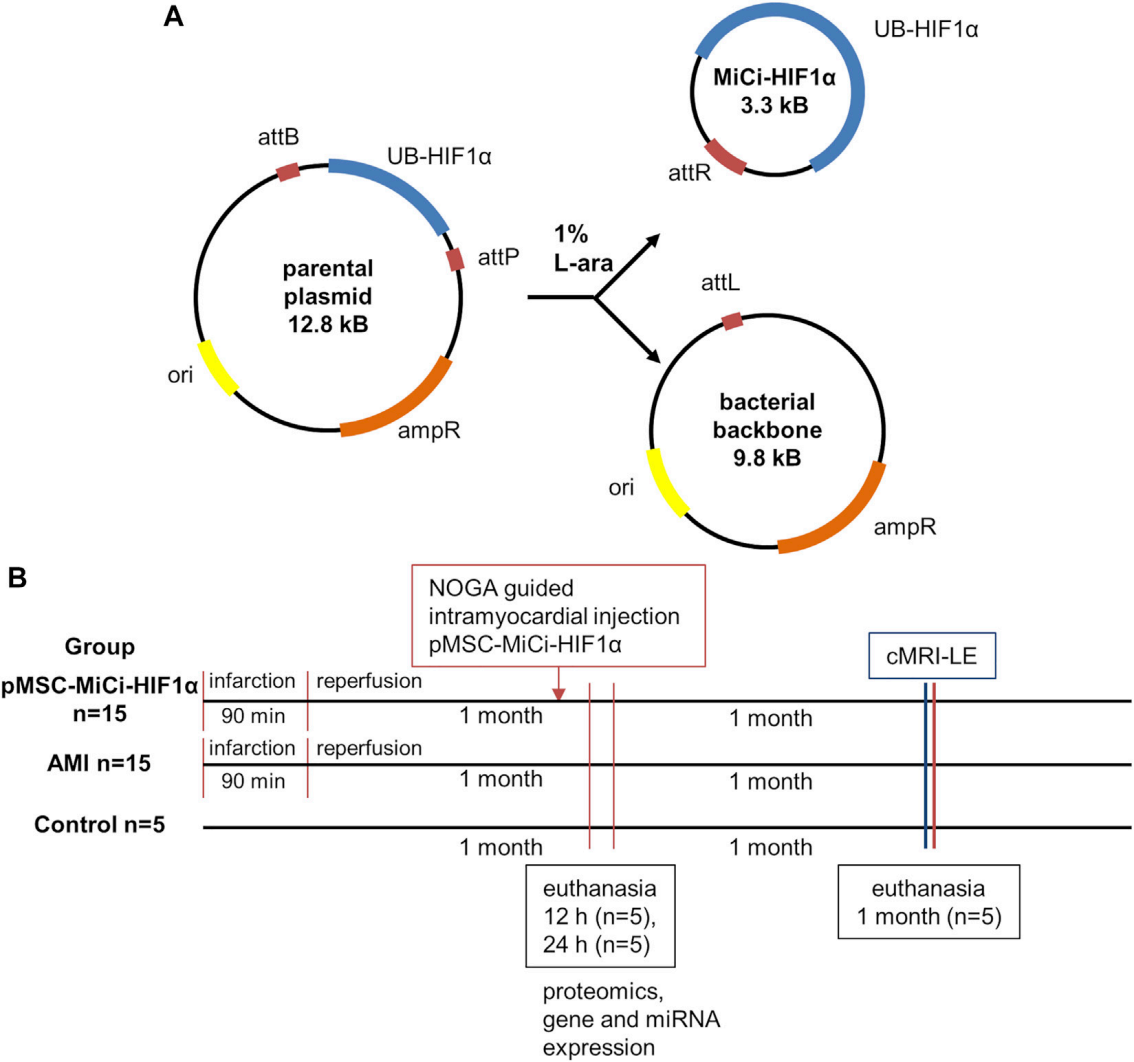
Minicircle structure and study design. **(A)** Characteristics of the minicircle HIF-1α (MC-HIF1α) expression vector. Upon bacterial expression, the MiCi-HIF-1α is produced by intramolecular recombination between the attB and attP sites. As a result, the expression vector carriers the transgene with the ubiquitin promoter (UB-HIF-1α) and the right hybrid sequence (attR). The bacterial backbone including the antibiotic resistance gene (ampR), the origin of replication (ori) and the left hybrid sequence (attL) is subsequently linearized and digested by nucleases. **(B)** Study design with indications of follow-up procedures and analyses. (cMRI-LE: cardiac MRI late enhancement, miR: micro-RNA, mRNA: messenger RNA, IHC: immuno-histochemistry).

Non-viral, non-integrating gene transfer through plasmid DNA has been widely employed *in vivo* and has resulted in transient and safe protein production (Jiang, Lee, and Pardridge 2020; Chong, Yeap, and Ho 2021; Paidikondala, Kadekar, and Varghese 2018). However, overall success in therapeutic settings has been hampered by frequent poor transfection efficiency and insufficient transgene expression, due to the use of rather large viral vectors for transfection. Minicircle (MiCi) vectors have been developed for overcoming these issues (Lijkwan et al., 2014; Narsinh et al., 2011; Jia et al., 2010). MiCi DNA is produced in bacteria from a recombinantly expressed parent plasmid by site-specific intramolecular recombination driven by bacteriophage integrase (Figure 1A). Being smaller than plasmid DNA, and lacking intergenic prokaryotic sequences, transfection into mammalian cells is facilitated and a longer duration of protein production can be achieved due to lack of binding of repressive proteins to the vector backbone (Krhac et al., 2017).

The detailed mechanism of action and individual characteristics of cell therapeutic efforts must be precisely understood to improve preclinical and translational results. Earlier studies discussed individual characteristics, cardiopoietic index and importance of careful patient selection for cardiac cell therapy. MSCs are easily available and known to have strong paracrine effects *via* secretion of a wide range of pro-angiogenic, anti-apoptotic and anti-fibrotic peptides and small molecules. (Fernandez-Aviles et al., 2017; Winkler et al., 2020). Novel therapeutic approaches must induce further paracrine factors to increase the therapeutic effects, and targeted genetic engineering might be a solution for enhancing cardiac repair mechanisms.

Hypoxia-induced factor 1-alpha (HIF-1α) is a master regulator of the cellular response to hypoxia and responsible for a number of downstream regulations at the transcriptional level, and corresponding functional effects. HIF-1α is stabilized under hypoxic conditions and consequently induces transcription of a multitude of target genes that are acting on metabolism, oxidative stress, cell cycle regulation, and others (Kimura et al., 2015; Kimura, Nakada, and Sadek 2017). HIF-1α has an essential role in protection against oxidative stress for regenerative capacity of a small subset of adult mammalian cardiomyocytes (Kimura, Nakada, and Sadek 2017). In adult mice, left ventricular ejection fraction (EF) was significantly improved after transfection of HIF-1α with MiCi carrier, compared to a standard HIF-1α plasmid (Huang et al., 2009). Co-delivery of an adenoviral vector encoding HIF-1α and MSCs enhanced cardiac function assessed by LVEF and fractional shortening in infarcted rats (Huang et al., 2014). The combined delivery of angiogenic factor HIF-1α embedded in MiCi and MSC showed beneficial effects such as improvement of cardiac function in an ovine ischemic myocardium model (Hnatiuk et al., 2016). HIF-1α is believed to be critical for maintenance and function of progenitor cells in hypoxic conditions such as in infarcted myocardium. As a central regulator, HIF-1α has been transduced into MSCs and resulted in improved cardiomyocyte proliferation, cardiac function, and a higher degree of angiogenesis compared to wild-type MSCs in infarcted rats (Cerrada et al., 2013).

Here we aimed to investigate the efficacy of cell-based regenerative therapy by engineering MSC with MiCi-HIF1-α vector, as a cell-based gene therapy in a translational large animal model of closed-chest reperfused acute myocardial infarction (AMI), and investigate further paracrine effects, such as proteome profile and expression of transcriptomics and miRs.

## Methods

### pMSCs Culture

pMSCs were isolated from bone marrow samples of healthy pigs by enzymatic dissociation and fluorescence activated cell sorting method (FACS) for CD90 and CD105 cell surface markers (Zlabinger et al., 2018). MSCs were cultured in plastic flasks (Sarstedt, Numbrecht, Germany) in standard MSC medium (Lonza, Basel, Switzerland) and passaged every 3–4 days with trypsin (Sigma-Aldrich, St. Louis, Missouri, U.S.) mediated enzymatic dissociation.

### Transfection of pMSCs With HIF1-α

pMSCs were transfected with a gene product consisting of HIF-1α enclosed in a MiCi backbone, a small circular plasmid for non-viral transgene delivery (MiCi-HIF1-α). Production and characteristics of the gene vector were described in detail previously (Huang et al., 2009). Several milligrams of MiCi-HIF-1α were produced in E.coli and isolated with Maxipreps (Qiagen, Hilde, Germany) according to established procedures (Hnatiuk et al., 2016). pMSCs were transfected with MiCi-HIF1α with lipofectamine in Optimem medium (Thermo Fisher Scientific, Waltham, Massachusetts, U.S.). For optimization of transfection conditions, a GFP-containing minicircle vector (Huang et al., 2009) was transfected into pMSCs (4,000 cells per well, 100–500 ng DNA/well)) in 24-well plates with 0.1–2.5 µL lipofectamine 2000. For *in vivo* transplantation, MSCs were seeded in 300 cm^2^ flasks (6 × 10^6^ cells per flask), incubated for 24 h, and transfected with 50 µg MiCi-HIF1α and 125 µL lipofectamine. The transfection was repeated three days after the initial transfection to improve efficiency and cells were incubated for further four weeks. Cell aliquots were lysed after 7, 14, and 28 days for western blot analysis (see 2.8). Then, pMSCs were trypsinized, re-suspended in 10 ml cell culture medium per flask, counted, collected by centrifugation and re-suspended in Cryostor medium at a density of 5 × 10^6^ cells per ml. An aliquot was lysed in RIPA buffer and successful MiCi-HIF1α transfection was verified by Western blot (pMSC-MiCi-HIF1α).

### Experimental Model of Closed-Chest Reperfused Myocardial Infarction

Animal care and handling followed the Guide for Care and Use of Laboratory Animals, published by the U.S. National Institutes of Health. Experimental protocols were approved by the Animal Use and Care Committee and by the Ethics Committee on Animal Experimentation at the University of Kaposvár, Hungary; in a Good Laboratory Practice-certified institute. Domestic adult female pigs received general anaesthesia as described earlier (Winkler et al., 2020). Briefly, closed-chest reperfused AMI was developed *via* percutaneous balloon occlusion of the left anterior descending (LAD) coronary artery (Lukovic et al., 2019). We used the femoral arterial site for angiography. The LAD was occluded for 90 min, followed by reperfusion. This protocol models human ST segment elevation myocardial infarction and reperfusion *via* primary percutaneous coronary intervention (PCI) and post-ischemic left ventricular remodeling. The animals were divided into AMI groups: Group pMSC-MiCi-HIF1α (n = 15), Group AMI (n = 15); Group Control animals (n = 5) were sham operated.

### pMSC-MC-HIF1α Delivery in Chronic Myocardial Ischemia

One month post-AMI, pMSC-MiCi-HIF-1α were injected percutaneously, intramyocardially guided by the 3D NOGA navigation technology. The endocardial mapping and injection technology have been reported in detailed previously by our group (Gyöngyösi and Dib 2011; Pavo et al., 2014a). In total, 5 ml pMSC-MiCi-HIF-1α were injected endomyocardially, in aliquots of 0.3 ml each at 12–14 injection sites per animal; in total 1 × 10^7^ cells per animal were administered.

Cardiac magnetic resonance imaging with late enhancement (cMRI-LE) one month post pMSC-MiCi-HIF-1α intramyocardial delivery.

One month post pMSC-MiCi-HIF1α delivery, the animals underwent cardiac magnetic resonance imaging (cMRI) with late gadolinium enhancement imaging to determine LV function, remodeling, and infarct size. The acquisition technique, imaging and analysis have been described previously (Pavo et al., 2014a; Pavo et al., 2014b). The LV end-diastolic and end-systolic volumes (EDV and ESV, respectively) were measured, and the LV stroke volume (SV), ejection fraction (EF) and cardiac output (CO) were calculated. Myocardial infarct size was measured from the LE imaging, as described previously (Pavo et al., 2014a; Winkler et al., 2020).

### Tissue Sampling

Animals at 12 and 24 h after pMSC-MiCi-HIF1a delivery (short term follow up, n = 5 in each group and each follow-up time) were harvested, myocardial fresh frozen samples were immediately collected in liquid nitrogen and stored at −80°C, in RNAlater (Sigma-Aldrich). Surviving pigs (n = 5) were followed-up for up to 1 month after pMSC-MiCi-HIF-1α delivery with equal follow-up periods for Group AMI and Group Control animals. Figure 1 shows the study design.

### Protein Isolation

*In vitro* pMSCs cultures (naïve and MiCi-HIF1α transfected) and fresh frozen myocardial tissue samples harvested from treated and control (healthy) pigs were used for proteomics analyses. Myocardial tissues were dissociated in a gentleMACS dissociator with M tubes (Miltenyi Biotec, Bergisch Gladbach, Germany). Dissociation steps followed the manufacturer’s protocol. Protein concentrations were determined with Coomassie (Bradford) Protein Assay Kit (Thermo Fisher Scientific). Colorimetric absorbance from Coomassie reaction was obtained at 540 nm in a microplate reader (1420 VICTOR3, Perkin Elmer, Waltham, Massachusetts, U.S.) and protein concentrations were calculated using bovine serum albumin standard curves.

### Western Blot

For Western blot (WB) analyses 50 µg proteins/sample were used either from cell culture (naive MSCs and after 7, 14, and 28 days after MiCi-HIF1α transfection) or from myocardial samples. Samples were loaded and separated on 7.5% acrylamide gels. After electrophoresis, proteins were blotted on PVDF membrane (#162–0261; Bio-Rad Laboratories, Hercules, California, U.S.) and stained with internal control *ß*-actin (Sigma-Aldrich, A2066, dilution 1/2000) and anti-HIF1α antibody (Abcam #51608, dilution 1/1000). As secondary antibody, goat anti-rabbit IgG (Abcam #6721, dilution 1/2000) was used. As a marker, a pre-stained Pageruler Standard was used (Thermo Scientific #26616). The blots were incubated with a luminol detection reagent (Thermo Fisher) and visualised on a Chemi-Smart system (Vilber Lourmat, Collégien, France) with optimised exposure times.

### Angiogenesis Proteome Profiling

Angiogenesis proteome profiling was carried out with 100 µg protein per sample (before cell injection, and 12 and 24 h as well as one month later) on Proteome Profiler Human Angiogenesis Array Kit (R&D Systems ARY007). Protein lysates were isolated as described above. Myocardial protein isolates were handled as described in the protocol guide. Chemiluminescent signal pixel densities were detected on X-ray films (Bio-Rad Molecular Imaging System) and analysed by ImageJ software (developed by the Laboratory of Optical and Computational Instrumentation, University of Wisconsin, NIH, U.S.). Myocardial tissue proteome was further analysed in the String functional protein association network database for Sus scrofa species (Szklarczyk et al., 2015). To assess relevance in human cardiac regeneration, the same protein library was analysed in Ingenuity Pathway Analyses.

### qRT-PCR

Total RNA, including small RNAs, was isolated from samples stored in RNAlater using a miRNeasy kit and the QIAcube system (Qiagen) according to the manufacturer’s instructions. For circulating RNA, the QIAamp Circulating Nucleic Acid kit was used. cDNA was prepared from 500 ng RNA *via* QuantiTect Kit (Qiagen), according to the manufacturer’s instructions. For qPCR measurements QuantiNova SYBR Green PCR Kit (Qiagen) and an ABI 7500 PCR cycler were used. The following transcriptoms were assessed: Angiopoietin, apelin, CD31, VEGF, HIF-1α, and CXCL12. miRNAs were reverse transcribed using the miScript II RT kit and quantified by qPCR with a miRNA specific and the Qiagen universal primer. The mRNA and miR primers are listed in Supplementary File.

### Immunohistochemistry

Myocardial samples were preserved in formaldehyde, embedded in paraffin, 3 µm slices were deparaffinized, permeabilized and prepared for immunohistochemistry staining. As primary antibody, anti-caspase-3 (Abcam #13847, dilution 1/100) was used. The secondary antibody was goat anti-rabbit IgG, DyLight 488 labelled (Abcam #96899, dilution 1/200). Cell nuclei were stained with Hoechst (Thermo Fischer #H3570, dilution 1/5000). Sections were captured in Olympus IX83 microscope and fluorescence intensities were analysed by ImageJ software.

### Statistical Analyses

Data are presented as mean ± standard deviation of mean (SD). T-test was used for comparing two groups, and one-way ANOVA with Bonferroni post-hoc tests were performed for multiple groups comparisons. Statistical significance is indicated at *p* ≤ 0.05. Statistical analyses were completed with GraphPad Prism v.7 (San Diego, California, U.S.).

## Results

### Study Design for Clinical Translation

The study protocol was designed with reasonable translational potential to human clinical applications, considering the 3R rules and following the ARRIVE guidelines. Experiments were approved by the ethics committee of the University of Kaposvar. Study design is shown in Figure 1B.

### Cell Transfection and Injections

**FIGURE 2:**
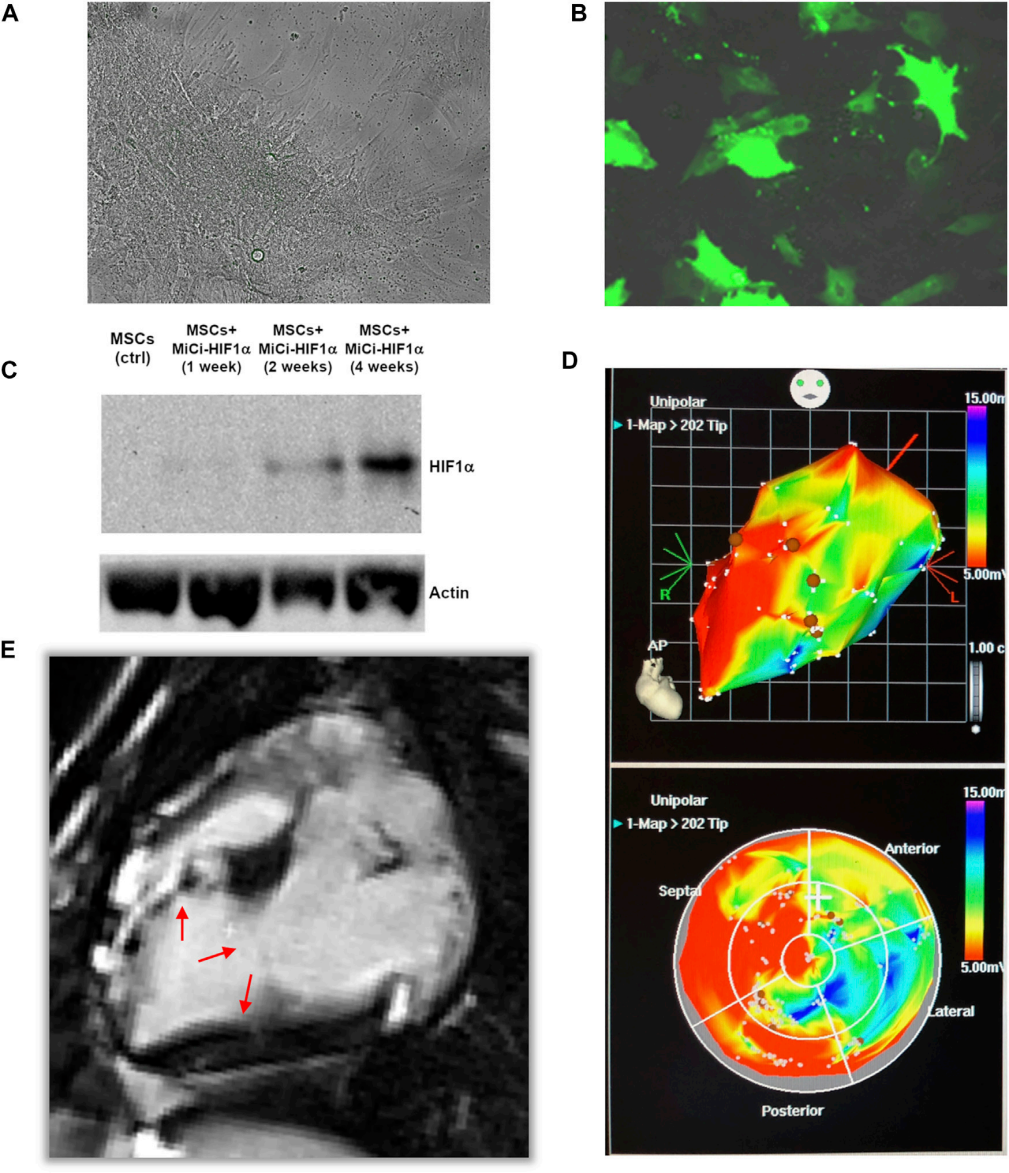
Minicircle-HIF1α transfection in pig mesenchymal stem cell. **(A)** Representative bright field picture of pig mesenchymal stem cell culture (MSC) before transfection of minicircle-HIF1α (MiCi-HIF1α). **(B)** Representative figure of transfected MSC culture, labelled with MiCi-HIF1α -green fluorescence protein 4 weeks after transfection. **(C)** Western blot showed gradually increased HIF1α protein expression in cell culture lysates (molecular weight 120 KDa) one, 2, and four weeks after the initial of two transfections. **(D)** 3D (upper) and 2D (bottom) NOGA electro-anatomical mapping and 3D-guided percutaneous intramyocardial injections (brown points). **(E)** Representative fcMRI (late-gadolinium enhancement) image 2 months after AMI and 1 month after cell injections. Arrows show the infarct border zone.

pMSC were transfected *via* a virus-free method for transient production of HIF1α. Transfected and non-transfected pMSCs were cultured under standard conditions (Figures 2A,B), and WB analyses of cellular lysates up to four weeks after the initial of a dual transfection proved successful, efficient, and long-lasting expression of HIF-1α (Figure 2C). Cell transfection efficiency was 63 ± 9% based on fluorescent microscopy using a GFP-expressing vector. This was in line with a 70–80% efficiency quantified by flow cytometry for the same minicircle vector for transfection of ovine MSCs. Myocardial infarctions were induced in pigs using a well-established closed-chest protocol (Zlabinger et al., 2018). All animals survived the surgical procedure and the treatment, and were followed up to the predefined final analyses. After 1 month, in the period of chronic ischemic left ventricular dysfunction, the pMSC-MiCi-HIF-1α were implanted into the border zone of infarction *via* electromagnetic guidance of NOGA percutaneous intra-myocardial biologics delivery system, as described previously (Gyöngyösi and Dib 2011; Pavo et al., 2014b). Implantation sites avoided necrotic myocardium Figure 2D), and cells were delivered into the border zone of infarction (Figure 2E). This ensures optimal and uniform delivery of stem cells to the target area.

### MiCi-HIF-1α Activates Angiogenesis and Remodeling-Related Proteins

**FIGURE 3:**
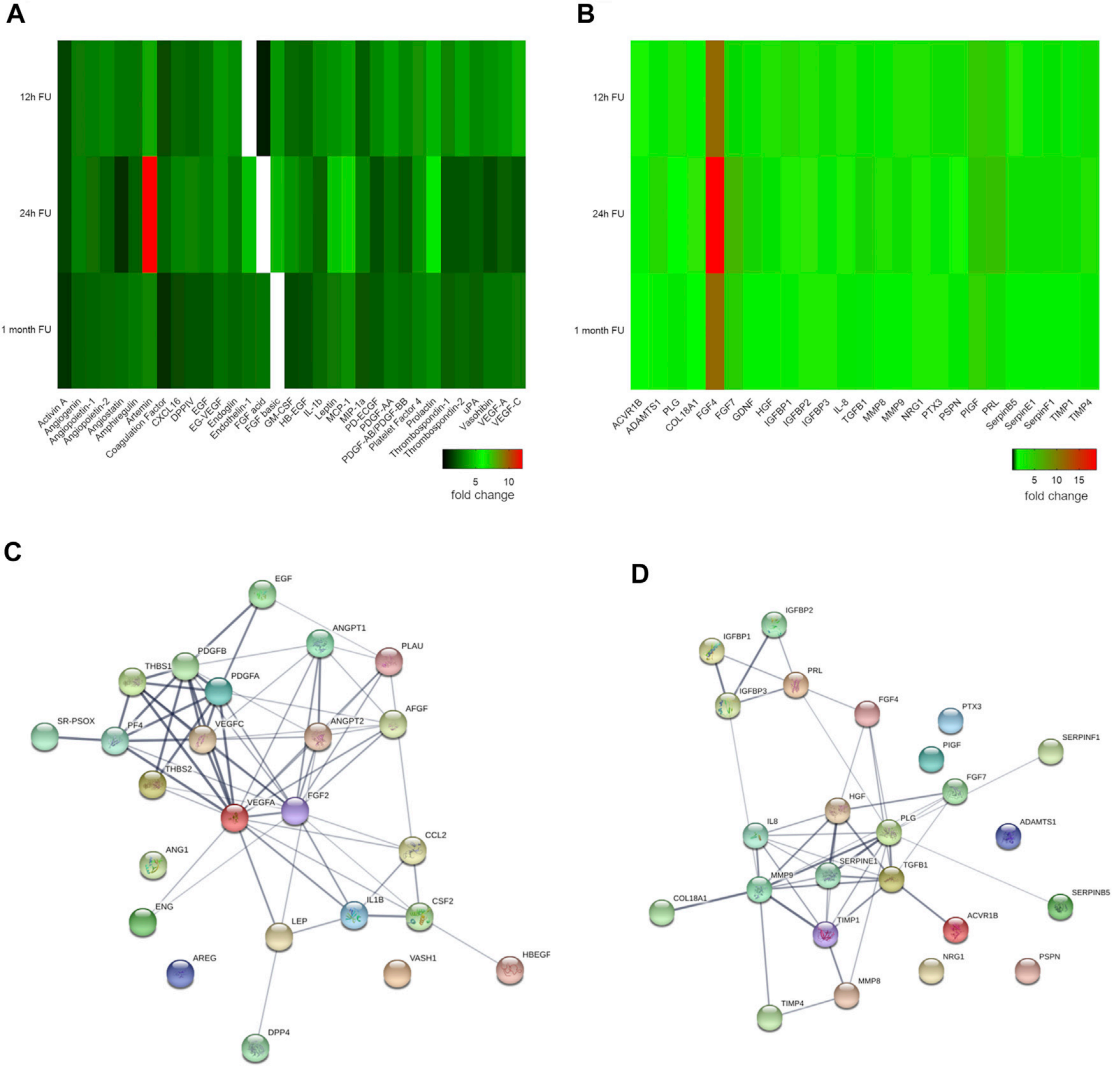
Angiogenesis and cell-matrix adhesion proteome profile. Heat maps show **(A)** angiogenesis and **(B)** cell-matrix adhesion related protein expression at 12 h, 24 h, and 1 month after injection of pMSC-MiCi-HIF1α. Protein levels are normalised to those in baseline samples (i.e. before cell injections), with heat map data representing relative changes according to the color legend. **(C,D)** String protein network analyses show functional protein interactions of the investigated proteome in Sus scrofa, irrespective of quantitative changes of the individual proteins. Line thickness indicates the degree of confidence of the interaction, based on curated experimental data, predictions from genomic context analysis, and text mining. Node colors are merely for illustration purposes. *n* = 3 biological replicates for short term pMSC-MC-HIF1α follow up, *n* = 3 biological replicates for control infarct hearts.

Proteome profiling of angiogenesis cell-matrix adhesion, and remodeling related proteins from myocardial lysates proved strong modification of the myocardial proteome in response to pMSC-MiCi-HIF-1α implantation (Figures 3A,B). Proteome profiling was performed at control, and at early (12 h, 24 h), and late time (1 month) points of follow up. Values were expressed relative to baseline, i.e., before cell implantation in order to selectively detect changes elicited by pMSC-MiCi-HIF1α. Major changes in protein abundance presented already early (12 and 24 h) after implantation, and were sustained at the one-month follow-up. These results suggest a robust paracrine activation in response to pMSC-MiCi-HIF-1α implantation compared to the control group. Among the most abundant angiogenesis proteins were activin A, angiopoietin, amphiregulin, artemin, CXCL16, DPP-4, endothelin-1, MCP-1; and remodeling factors ADAMTS1, FGFs, TGFb1, MMPs and serpins. We found significant increase of DPP-4, which is a key factor in sprouting angiogenesis, promotes peri-cellular proteolysis and enhances endothelial cell migration and invasion into surrounding ECM. Amphiregulin is a ligand of endothelial-growth factor receptor, which favors neo-angiogenesis. Artemin can attract hematopoietic and angiogenic precursor cells and activate them. CXCL16 is a scavenger receptor activator for macrophages and involved in atherosclerotic and neo-angiogenic processes. Endoglin is a key glycoprotein on endothelial cells and involved in angiogenesis. FGF-1 and FGF-2 are important regulators of endothelial cell development and they are key in angiogenesis regulation.

In terms of cell-matrix adhesion and remodeling-related proteins, ACVR1B was found in higher abundance, suggestive of activation of mechanisms related to ECM production and wound healing, furthermore it also counteracts with angiogenesis. FGF-4 and FGF-7 are key regulators of cellular proliferation in various tissues, predominantly during embryonic development. ADAMTS isoforms are involved in matrix degradation and disintegration. Besides regulation of clot degradation, plasminogen also acts on tissue remodeling, having strong catalytic activity. IGFBPs promote cell migration and smooth muscle cell survival. Collagen XVIII showed elevated levels at early follow-up periods, suggestive of rapid deterioration of extra-cellular matrix in infarcted myocardium.

Detailed analysis of the protein network was done using the String database (Szklarczyk et al., 2015) for Sus scrofa species. VEGFs, angiopoietin-2 and thrombospondin were revealed as the driver components of angiogenesis networks, showing strong evidence for interaction (Figure 3C). Within the matrix remodeling network, MMP9, TGFB1, and SERPINE1 had pivotal roles (Figure 3D). To assess the relevance of these proteins in humans, an ingenuity pathway analyses of the activated protein networks was performed, corroborating the important role of the identified protein network in a number of key functional cardiovascular mechanisms (Supplementary Figure S1).

### pMSC-MiCi-HIF1α Implantation Initiated Angiogenic Changes in Gene and miRNA Expression

**FIGURE 4:**
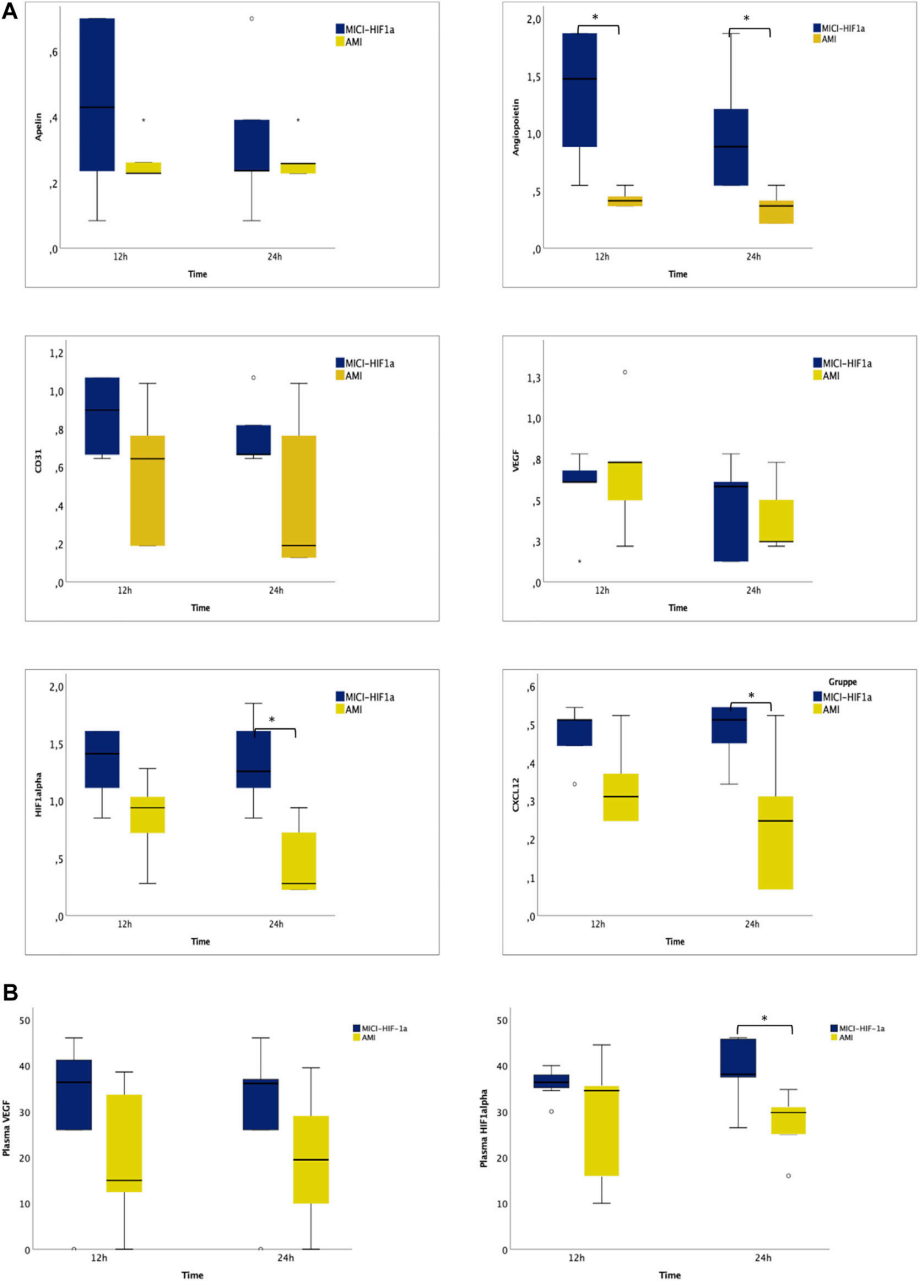
Myocardial expression of angiogenesis and remodeling related genes, and circulating RNAs in plasma 12 and 24 h after intramyocardial injection of porcine MSC transfected with MiCi-HIF1α into the border area of chronic infarction, compared to non-treated infarcted heart. **(A)** mRNA expression levels of apelin, angiopoietin, CD31, VEGF, HIF-1 *a,* and CXCL12 in pMSC-MICI-HIF-1α treated and untreated myocardium, 12 and 24 h after injection of pMSC-MiCi-HIF1α, data are represented relative to healthy non-infarcted controls (relative expression 1.0 for each) with normalization to the reference gene *ß*-actin. *n* = 5. FU: follow up. Statistics: t-test, *: *p* <0.05. **(B)** Circulating mRNA levels of VEGF and HIF-1 *a* in pMSC-MICI-HIF-1α treated and untreated animals, 12 and 24 h after injection of pMSC-MiCi-HIF1α. Data are represented as relative expression. *n* = 5. *: *p* <0.05.

To complement the investigations, we examined the expression of selected relevant angiogenic genes 12 and 24 h after pMSC-MiCi-HIF-1α delivery, compared to healthy control animals and untreated AMI (Figure 4). Angiogenic factor angiopoietin expression increased at the earlier time point of 12 h, and remained elevated at the 24 h follow-up. HIF-1α expression was also elevated at 12 h, but reached a significance at the 24 h follow-up, similar to the stem cell homing chemokine CXCL12.

For detection of systemic effects, we measured circulating RNA levels of VEGF and HIF1α. Significant elevation of circulating HIF1α mRNA was detected 24 h after cell injection.

**FIGURE 5:**
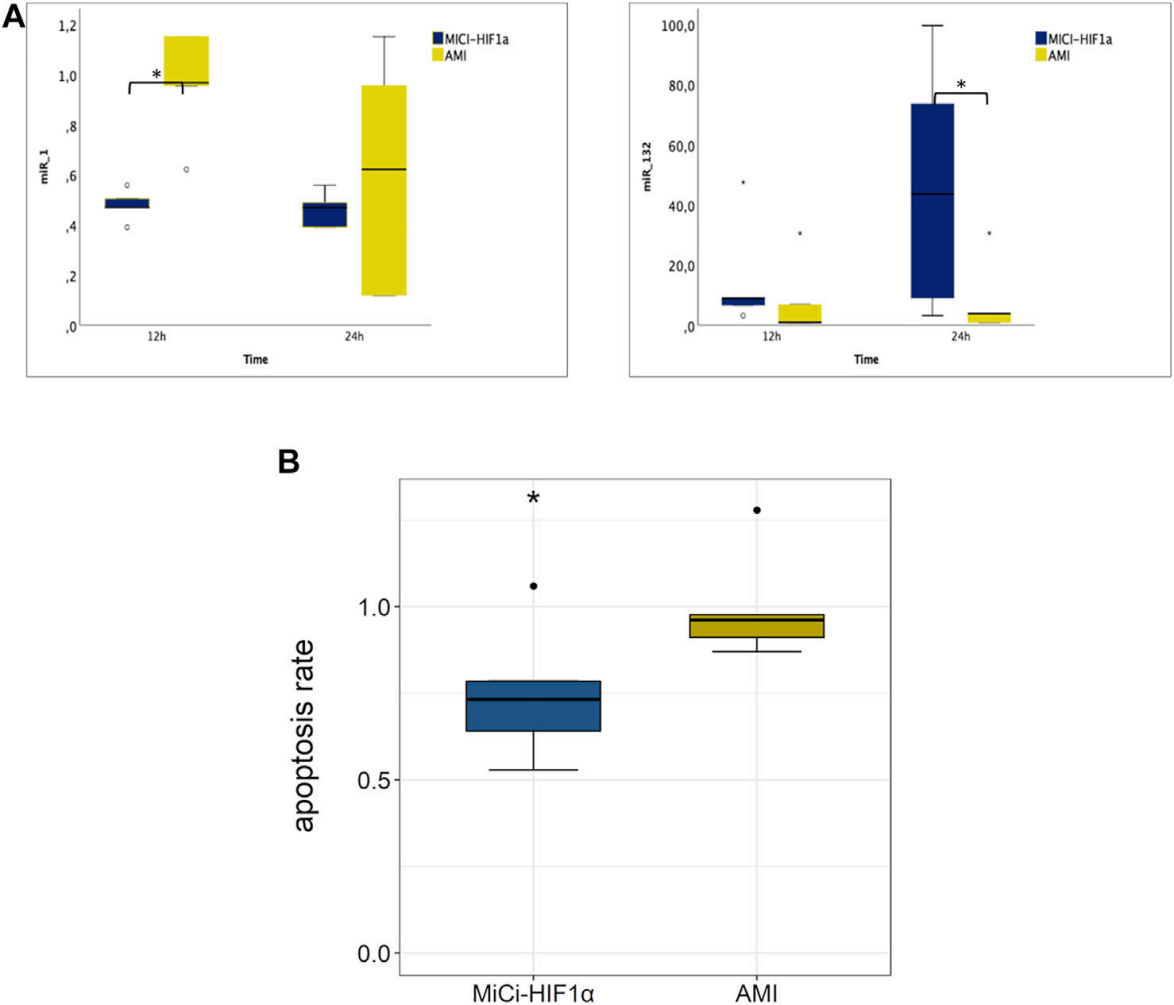
Myocardial expression of miRNAs in plasma 12 and 24 h and apoptosis 1 month after intramyocardial injection of porcine MSC transfected with MiCi-HIF1α into the border area of chronic infarction, compared to non-treated infarcted heart. **(A)** miR-1 and miR-132 expression levels are represented relative to healthy controls (relative expression 1.0 for each) with normalization to the reference let7a. Myocardial samples were collected from the border zone of infarction 12 and 24 h after injection of pMSC-MiCi-HIF1α *n* = 5. Statistics: test-test, *:*p* <0.05. **(B)** Relative changes in apoptosis rate in pMSC-MiCi-HIF1α treated animals compared to those without treatment at the 1-month follow-up. Apoptosis was assessed by determination of caspase-3 in histological slices. *n* = 5. Statistics: t-test, **: *p* <0.01, *: *p* <0.05.

Changes in miRNA expressions were also assessed from the border zone of the infarction at 12 and 24 h delivery of MSC-MiCi-HIF-1α. We investigated levels of miR-1 and miR-132, which are cornerstones in the regulation of cardiac remodeling and angiogenesis (Figure 5A). Anti-hypertrophic miR-1 levels were significantly increased in the infarcted border zone without cell implantation at the 12 h follow-up. miR-1 is a muscle-specific miRNA with distinct expression in cardiomyocytes (Karakikes et al., 2013). It has been proposed as a circulating biomarker, as its plasma concentration is elevated after AMI, arguably caused by necrosis of cardiomyocytes (Cheng et al., 2010). miR-1 expression in the heart is reduced in the infarcted area but has been found to be increased in border and remote zones of the myocardium (Bostjancic et al., 2010). Furthermore, angiogenic miR-132 levels showed highly elevated expression 24 h after pMSC-MiCi-HIF1α delivery. Immuno-histochemistry analyses of myocardial samples at 1 month follow up proved decreased apoptosis rate in response to pMSC-MiCi-HIF1α treatment (Figure 5B).

### CMR Results

**FIGURE 6:**
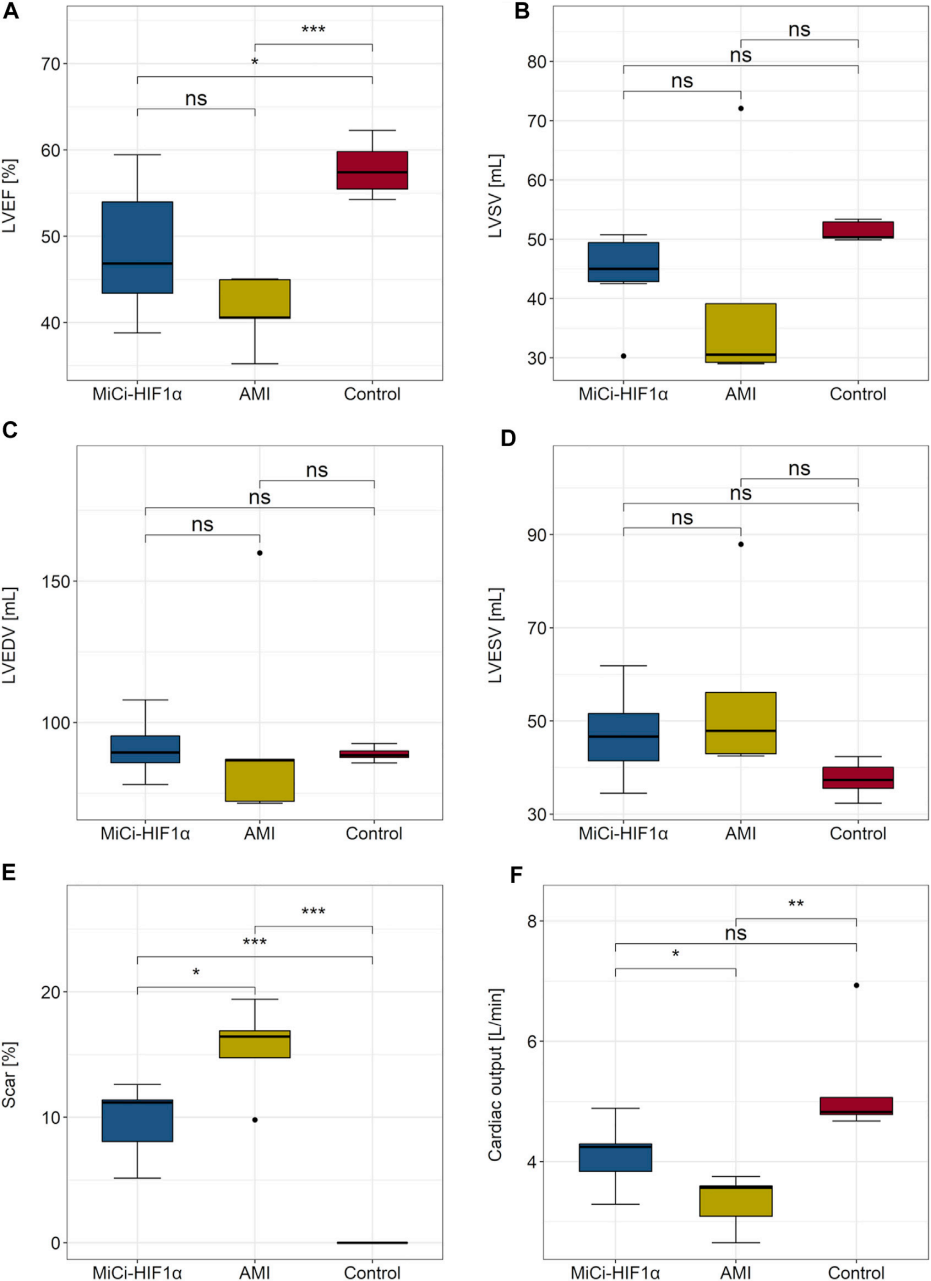
Cardiac magnetic resonance imaging (cMRI) assessment of functional cardiac parameters. cMRIwith late gadolinium enhancement was performed 2 months afterinfarctionandone-month post pMSC-MiCi-HIF-1α percutaneous intramyocardial delivery.Results are shown in boxplots with dots representing outliers.**(** **A)** Left ventricular (LV) ejection fraction (EF), **(B)** LV systolic volume (SV), **(C)** LV end-diastolic volume (EDV), **(D)** LV end-systolic volume (ESV), **(E)** Myocardial infarct size, expressed as % of the LV, **(F)** Cardiac output (CO). Statistical significance was assessed by ANOVA and Bonferroni post-hoc test. ***: *p*<0.001. **: *p*<0.01, *: *p*<0.05, ns: not significant.

Figure 6 displays the cMRI results 2 months after AMI and one-month post pMSC-MiCi-HIF-1α percutaneous intramyocardial delivery. Treatment with pMSC-MiCi-HIF-1α improved cardiac output (4.1 ± 0.5 vs. 3.3 ± 0.4 L/min) and reduced the myocardial scar size (9.8 ± 2.7 vs. 15.5 ± 3.2%) compared to the untreated AMI group. We detected no significant change of the left ventricular ejection fraction (LVEF) after pMSC-MiCi-HIF-1α treatment compared to untreated animals (48.4 ± 7.2 vs. 41.3 ± 3.6%, *p* = 0.13).

### Limitations

A limitation of this study is the small number of sacrificed animals at baseline and short term follow up (12 h, 24 h) for analysis of angiogenetic proteins and transcriptomics, to apply the 3R rules (reduce, replace, refine). Longitudinal proteome and transcriptional analyses were not possible. Side-by-side comparison of pMSCs and pMSC-MiCi-HIF-1α implantation was also not feasible in keeping with 3R rules. Here, we refrained from following the biodistribution of MSCs, since respective data has already been shown elsewhere (Gyöngyösi et al., 2008; Pavo et al., 2014b; Zlabinger et al., 2018), and the precise injection sites cannot be easily identified more than 24 h later. In those studies, even though approx. 90% of implanted MSCs were eliminated from the injection site within 24 h, the remaining cells can be expected to express therapeutically meaningful amounts of HIF1α.

We aimed to achieve a significantly better cardiac performance as a response to the cell-based cardiac gene therapy. The LVEF was improved (48.4 ± 7.2 vs. 41.3 ± 3.6%, *p* = 0.13), but did not reach statistical significance. To note, the number of the animals was kept at the minimum. Thus, the study might be underpowered for detecting an effect of such a difference. Nevertheless, an approximately 7% difference in LVEF between the groups at the final MRI is remarkable; as improvement of EF ≥5% as a response to therapy is clinically relevant, as defined in several cardiac regeneration studies (MYSTAR, EUROINJECT (Gyöngyösi et al., 2005; Gyöngyösi et al., 2009)). Additionally, HF patients with EF <45% are included in clinical regeneration studies as a need for cardiac regeneration MSC-HF (Mathiasen et al., 2020), or on-going SCIENCE (Paitazoglou et al., 2019)); accordingly, the animals in the pMSC-MiCi-HIF-1α have reached a level of therapeutic efficacy. In accordance with the current guidelines, that HF with mildly reduced EF (HFmrEF) is characterized as HF with an LVEF of 41–49%. (Bozkurt et al., 2021), the LV EF of the animals of the group pMSC-MiCi-HIF-1α is at the upper border, and the LV EF of the animals in the untreated AMI group is at the bottom border of the HFmrEF definition. Of note, we did not find changes in LV volumes (end-diastolic and end-systolic) though cardiac LVEDV counts as a key prognostic factor after ischemic events (Reindl et al., 2019). We explain the slightly improved LVEF with the decreased scar size measured on LGE images. CMR image acquisition in this porcine model limited the availability of mapping techniques (especially T1) to better understand the mechanisms behind.

cMRI with late gadolinium enhancement was performed 2 months after infarction and one-month post pMSC-MiCi-HIF-1α percutaneous intramyocardial delivery. Results are shown in boxplots with dots representing outliers. A. Left ventricular (LV) ejection fraction (EF), B. LV systolic volume (SV), C. LV end-diastolic volume (EDV), D. LV end-systolic volume (ESV), E. Myocardial infarct size, expressed as % of the LV, F. Cardiac output (CO). Statistical significance was assessed by ANOVA and Bonferroni post-hoc test. ***: *p* <0.001. **:*p* <0.01, *:*p* <0.05, ns: not significant.

## Discussion

The hypoxia-induced HIF-1α acts on a plethora of downstream targets and transcription factors for counteracting detrimental effects of hypoxia. Here we present 1) safe and efficient transfection with a short circular expression vector, a MiCi encoding the human HIF-1α protein under control of the ubiquitin promoter; 2) efficacy of long-term transcription of HIF-1α and related angiogenic proteins; 3) and pre-clinical efficacy of this cell-based gene therapy to decrease ischemic burden of the left ventricle and improving hemodynamics in a translational model of chronic ischemic left ventricular dysfunction.

Recently many studies have proposed that implanted repair cells are not sustained in the myocardium, fail to couple electromechanically, and are not transplanted in sufficient numbers; thus, they overall lack reasonable regenerative capacity as shown in clinical trials (Fernandez-Aviles et al., 2017). Retention rate of bare pMSCs in the ischemic cardiac tissue has been shown insufficient and efforts initiating homing of exogenous cells with regenerative capacity, such as culturing the cells in hypoxic medium or pretreatment with cell adhesion molecules failed yet (Pittenger et al., 2019). Paracrine and immunomodulatory effects are thought to be essential for effective cell-based cardiac repair therapy, and thus genetically engineered stem cells might be promising. The novelty of our cardiac regenerative approach is the non-viral transfection of the cells with anti-ischemic HIF-1α with sustained HIF-1α expression. Even if the duration of the MSCs homing in the myocardium is short, a longer graft persistence of the MSCs in the myocardium is not excluded, although it was not possible to follow up the fate of MSCs *in vivo* in detail. The increase in circulating levels of HIF1α mRNA might be indicative that a proportion of MSCs is washed out of the myocardium. Circulating levels of angiogenesis genes such as VEGF were unchanged between treated and untreated animals. Therefore, a systemic angiogenetic effect was not detected up to 24 h after cell injection. Additionally, a remote paracrine effect of the migrated and extracardiac deposited MSCs exerting cardiac regeneration is also proposed (Peng et al., 2016). Animals underwent treatment one month after infarction, which more aptly reflects realistic clinical application, but arguably complicates successful outcomes, because the initial repair and immunological processes start immediately at the onset of infarction. Nonetheless, pMSC-MiCi-HIF-1α significantly reduced myocardial scar size and improved cardiac output. Our results thus underline the potential as a therapeutic concept. For mechanistic investigations, we focused on the short- and long-term paracrine effects of MSC-MC-HIF1α transplantation and assessed the concentration of angiogenetic proteins, transcriptomics and changes of miR expression on failing hearts of ischemic aetiology. The protein array allows screening of a set of proteins. While changes in single proteins in absence of validation should be interpreted with caution, the overall response clearly indicates a concerted activation of angiogenetic processes and cell-matrix adhesion related protein secretion. HIF-1α is well recognized as a master regulator of angiogenesis, (Zimna and Kurpisz 2015), and our results show a profound increase of a number of proteins involved in angiogenesis upon pMSC transplantation. This indicates a concerted regulation through the well documented role of HIF1α as a transcription factor, acting directly or indirectly on the proteins investigated in this study. Proteins related to cell-matrix adhesion have major roles in adverse remodeling during and after ischemic myocardial injury. The proteins with the strongest increase in the myocardial samples after 24 h are the growth factors FGF-4 and artemin. Fibroblast growth factors such as FGF-4 have, among other functions, profound endo- and paracrine effects on endothelial growth. Artemin is a neurotrophic factor belonging to the TGF-β signaling superfamily that is expressed in smooth muscle cells. It promotes cell survival, proliferation, and development. (Ilieva et al., 2019). These two proteins are exemplary for the angiogenetic response, with their precise role to be investigated in follow-up experiments.

Arguably, direct transcriptional activation induced by the pMSC-MiCi-HIF-1α treatment is more pronounced in a short timeframe after MSC implantation, and leads to prolonged effect in terms of infarct size reduction. Effects were reflected by increased expression of angiopoietin already at 12 h after cell implantation, and were more pronounced after 24 h with significant expression of HIF-1α, angiopoietin, and the stem cell homing chemokine CXCL12. No significant expressional changes of the signaling factor apelin (Helker et al., 2020) and the endothelial progenitor cell marker CD31 were found at these points, indicating no strong role of these processes early after cell transplantation. A number of transcriptional factors are induced by HIF-1α, and in turn can regulate miRNA-expression. miR-1 plays a role in cardiac remodeling and represses the development of hypertrophy. In a pig model of hypertrophy and cardiac fibrosis, reduced miR-1 expression was found. (Gyongyosi et al., 2017). The pro-fibrotic miR-21 is predominantly expressed in cardiac fibroblasts and was found to be upregulated in infarcted mouse hearts and generally under hypoxic conditions (Yuan et al., 2017).

In conclusion, pMSC-MiCi-HIF-1α treatment improves cardiac output and reduces myocardial scar. Mechanistically, an enhancement of the secretion of pro-angiogenic and anti-remodeling proteins, transcriptomics and miR short term after implantation (up to 24 h follow up) was demonstrated. pMSC-MiCi-HIF-1α enhances the intrinsic paracrine effects of MSCs implanted in ischemic failing hearts. Results of the proteome showed a pivotal role of upregulated proteins in the human cardiovascular system. This scope gives our results a major translational potential.

## Data Availability

The original contributions presented in the study are included in the article/Supplementary Material, further inquiries can be directed to the corresponding author.
