# Supplementary material for: Cell-Based HIF1α Gene Therapy Reduces Myocardial Scar and Enhances Angiopoietic Proteome, Transcriptomic and miRNA Expression in Experimental Chronic Left Ventricular Dysfunction
Source: Front Bioeng Biotechnol. 2022 May 12;10:767985. doi: 10.3389/fbioe.2022.767985 (PMC9133350; doi:10.3389/fbioe.2022.767985)
Supplement: Supplementary file 1 [file Table1.DOCX]

**Supplementary Table 1. Primer sequences**

| Target | forward | reverse |
| --- | --- | --- |
| Angiopoetin-2 | AGAGACTGCGCAGAAGCATT | GTCGCAGTAAGCCTTGGTCT |
| Apelin | TATTTGGTGCAGCCCAGAGG | CCCCTTCAGTCCTGCTTCAG |
| CD31 | CCCCAAGGGAGTGATCATAG | TGTCACTTGAATGGTGCACTT |
| CXCL12 | GCCAACATCAAGCATCTCAA | AGAGAGTGGGACTGGGTTTG |
| HIF-1α | ACCTGAGCCTAACAGTCCCAGTG | TTCTTTGCCTCTGTGTCTTCAGCAA |
| VEGF | GCCCACTGAGGAGTTCAACA | ACACGTCTGCGGATCTTGTA |
| Actin | TCAACACCCCAGCCATGTAC | CTCCGGAGTCCATCACGATG |
| miR-1 | GCAGTCTGGAATGTAAAGAAG |  |
| miR-21 | CGTAGCTAGCTTATCAGACTG |  |
| let7a | GCAGTGAGGTAGTAGGTTGT |  |
