# Supplementary figures and images for: Cell-Based HIF1α Gene Therapy Reduces Myocardial Scar and Enhances Angiopoietic Proteome, Transcriptomic and miRNA Expression in Experimental Chronic Left Ventricular Dysfunction
Source: Front Bioeng Biotechnol. 2022 May 12;10:767985. doi: 10.3389/fbioe.2022.767985 (PMC9133350; doi:10.3389/fbioe.2022.767985)

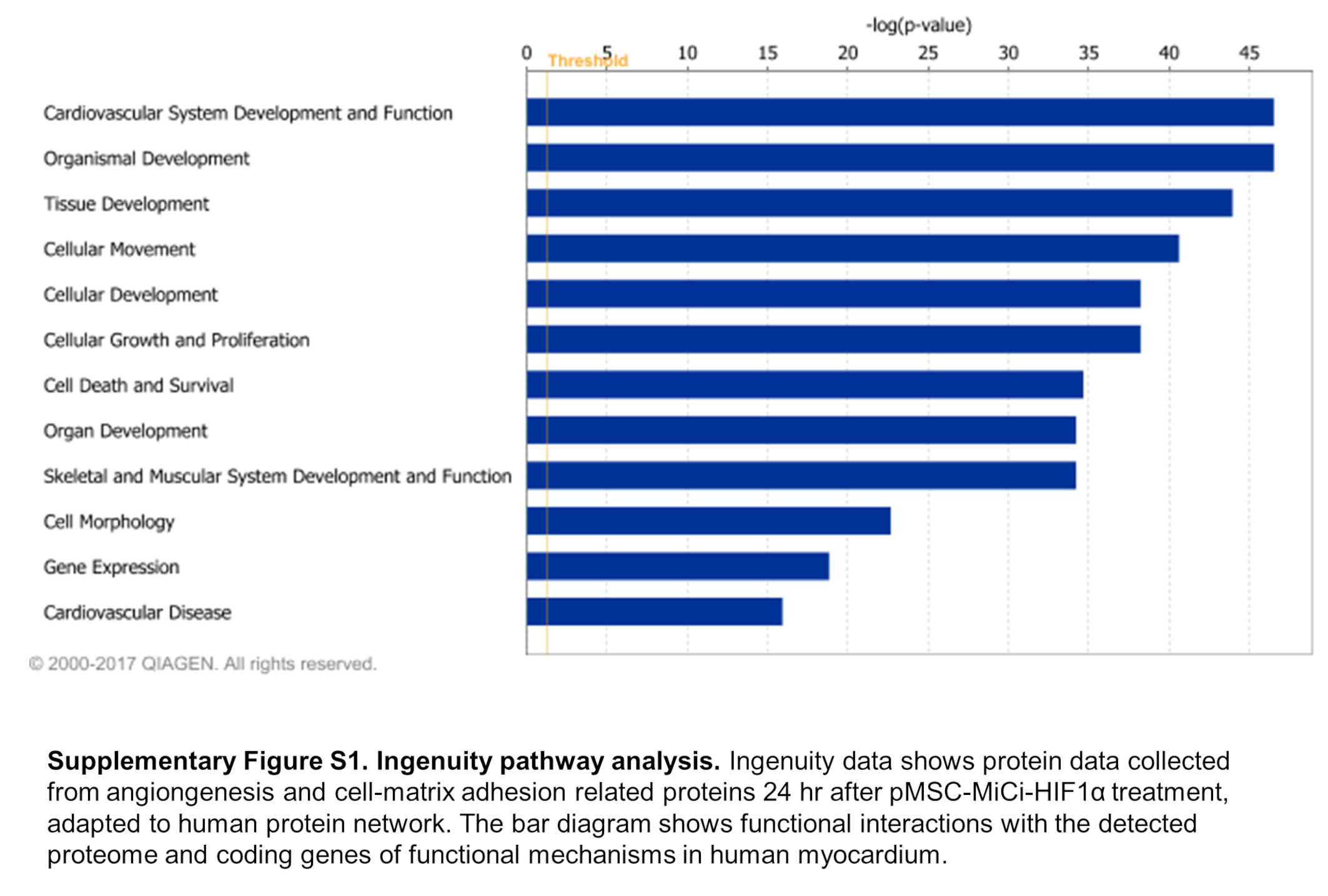

Supplement: Supplementary file 2 [file Image1.TIF]
